# Long noncoding RNA H19 suppresses cardiac hypertrophy through the MicroRNA-145-3p/SMAD4 axis

**DOI:** 10.1080/21655979.2021.2017564

**Published:** 2022-02-09

**Authors:** Hao Wang, Xiaoqing Lian, Wei Gao, Jie Gu, Haojie Shi, Yao Ma, Yafei Li, Yi Fan, Qiming Wang, Liansheng Wang

**Affiliations:** a Department of Cardiology, First Affiliated Hospital of Nanjing Medical University, Nanjing, China; b Department of Geriatrics, Sir Run Run Hospital of Nanjing Medical University, Nanjing, China

**Keywords:** Cardiac hypertrophy (ch), h19, miR-145-3p, smad4, molecular mechanism

## Abstract

Sustained cardiac hypertrophy (CH) contributes to many heart diseases. Long noncoding RNAs (lncRNAs) collectively play critical roles in cardiovascular diseases (CVDs). However, the roles of lncRNA H19 in CH are still unclear. A CH model was constructed utilizing isoproterenol (ISO). We demonstrated H19 could participate in regulating ISO-induced CH development both *in vivo* and *in vitro*. The online databases DIANA and TargetScan were used to predict the targets of H19 and MicroRNA-145-3p (miR-145-3p), respectively. Luciferase reporter assay was used to verify the downstream targets. The results showed that H19 was decreased under ISO stimulation. The H19 overexpression resulted in significant decrease in mouse heart size and weight, left ventricular systolic dysfunction, left ventricular posterior wall thickness and cardiac hypertrophic growth, while promoted the increase of left ventricular ejection fraction and left ventricle fraction shortening. H19 also inhibited protein expression levels of CH markers, such as atrial natriuretic peptide (ANP), brain natriuretic peptide (BNP), and MYH7. Luciferase assays results showed that miR-145-3p was a target of H19 and SMAD4 was a target of miR-145-3p. We found that H19 regulated SMAD4 by sponging miR-145-3p. Knockout of miR-145-3p or overexpression of SMAD4 facilitated H19-induced decreases in ANP, BNP, and MYH7. Collectively, our findings have indicated that the H19/miR-145-3p/SMAD4 axis should be a negative regulator involved in CH progression.

## Graphical abstract

**Figure:**
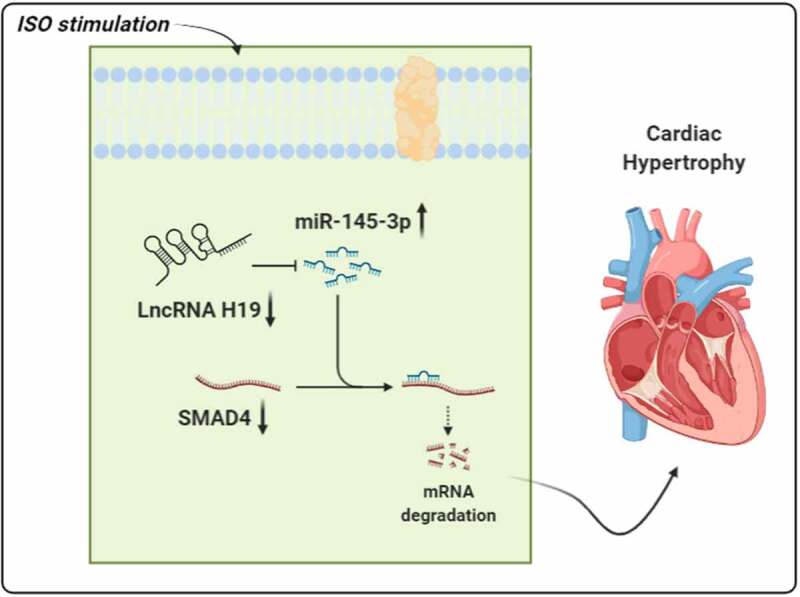

## Introduction

Cardiac hypertrophy (CH) is an early milestone of many heart diseases that occurs in response to various intrinsic or extrinsic stimuli [1]. CH is characterized by the enlargement of heart size, and cardiomyocyte surface area with no change in cell number [2]. However, sustained myocardial hypertrophy contributes to maladaptive ventricular remodeling and heart failure. Moreover, various factors complicate the therapy for CH, such as hyperglycemia, insulin resistance, activation of the renin-angiotensin-aldosterone system, inflammation, and oxidative stress [3]. Therefore, investigation of the specific mechanism involved in the CH development is of vital importance.

Long noncoding RNAs (lncRNAs) are transcribed RNAs >200 nucleotides in length without protein-coding functions, and they are key players in cardiovascular disease (CVD) [4]. Previous studies have revealed that aberrantly expressed lncRNAs contribute to the initiation and progression of CH [5]. LncRNAs regulate gene transcription and posttranscriptional processing and DNA damage, which are strongly involved in heart disorders [6]. For instance, terminal differentiation-induced ncRNA (TINCR) inhibits myocardial hypertrophy by silencing CaMKII [7]. Knockout of taurine upregulated gene 1 (TUG1) suppresses cardiac hypertrophy by sponging miR-29b-3p [8]. AK045171 protects against cardiac hypertrophy by activating the SP1/MG53 signaling pathway [9]. LncRNA H19, located on human chromosome 11, is an imprinted gene. It collectively participates in embryonic development and growth control. A previous study demonstrated that H19 plays a cardioprotective role by suppressing programmed necrosis [10]. The role of H19 in cardiac hypertrophy has been previously investigated in both mouse and pig models of cardiac hypertrophy in human samples and cell cultures [11]. Moreover, H19 is aberrantly expressed in cardiac remodeling [12]. A study has measured the circulating levels of H19 in the plasma samples from 300 patients with coronary artery disease (CAD) and 180 control subjects, which identified that plasma levels of H19 were increased in CAD patients with heart failure compared to those with normal cardiac function [13]. Therefore, H19 may play a crucial role in regulating heart function. However, the potential roles of H19 in CH are still not clear.

In recent years, miRNAs have attracted extensive attention in the field of cardiovascular disease [14]. A growing number of circulating miRNAs associated with cardiovascular diseases, including CAD, acute coronary syndrome (ACS), acute myocardial infarction (AMI), heart failure (HF) and atrial fibrillation (AF), have been recognized as novel biomarkers for early diagnosis and prognosis [15]. MiRNAs microarray analysis showed that the expression levels of miR-145-3p were significantly upregulated in patients with acute myocardial infarction (AMI) compared to the control group [16]. Our study was designed to explore the functions of miR-145-3p in CH.

SMAD4 is a crucial regulator of TGF/BMP signaling [17]. SMAD4 binds with its receptor to form receptor-activated SMADs (R-Smads) to degrade its targets in the maintenance of developmental and homeostatic processes [18]. In CVD, SMAD4 suppresses transforming growth factor-β (TGF-β1)-induced cell proliferation [19]. SMAD4 regulates endothelial cell function, and modulates vessel sprouting and remodeling, maintaining blood vessel remodeling, maturation, and integrity [20]. The disruption of SMAD4 contributes to CH and heart failure [21]. However, the potential roles of SMAD4 in CH have not been fully elucidated.

lncRNAs H19 is strongly involved in heart disorders and the role of H19 in cardiac hypertrophy has been previously investigated in mouse models, human samples and cell cultures. We wanted to find the mechanism of how H19 regulates CH. MiRNAs microarray analysis showed that the expression levels of miR-145-3p were significantly upregulated in patients with AMI compared to the control group. SMAD4 was predicted to be a target of miR-145-3p in our present study. We would prove that H19 suppresses cardiac hypertrophy through the MicroRNA-145-3p/SMAD4 axis.

## Materials and Methods

### Clinical samples

Clinical serum samples were collected from chronic heart failure (CHF) patients diagnosed at the First Affiliated Hospital of Nanjing Medical University Hospital from May, 2017 to July, 2019. The inclusion criteria was defined according to the general inclusion criteria specified by the European Society of Cardiology [22], which was follows: 1) patients with CHF; 2) patients with complete medical records; 3) patients who provided informed consent. Additionally, healthy volunteers were recruited. Blood samples (5 *ml*) were obtained by venipuncture with the informed consent of volunteers. The study was approved by the Ethics Committee of First Affiliated Hospital of Nanjing Medical University Hospital. Each patient signed the informed consent. Patient information was listed in the supplementary materials Table 1.

**Table 1.** Basic information of the subjects in the study

Characteristics	CHF	Normal	*P*
	N = 30	N = 30	
Age, yrs	64.9 ± 3.3	65.0 ± 3.2	0.430
Sex, Male n (%)	16 (53.2)	17 (56.7)	0.535
LVEF (%)	33.4 ± 5.9	61.6 ± 7.1	<0.001
Pro-BNP (pg/mL)	3172 ± 552	229 ± 48	<0.001
Treatments n (%)			
ACEI/ARBs/ARNI	29 (96.7)	12 (40.0)	<0.001
β-blocker	27 (90.0)	9 [5]	<0.001
Spirolactone	25 (83.3)	2 (6.7)	<0.001
Digoxin	3 (10.0)	0 (0)	0.237
SGLT-2i	14 (46.7)	2 (6.7)	<0.001

CHF, chronic heart failure; LVEF, left ventricular ejection fraction; ACEI, angiotensin-converting enzyme inhibitor; ARB, angiotensin receptor inhibitor; ARNI, angiotensin receptor-neprilysin inhibitor.

### Animal care and use

Male C57BL/6 J mice aged 8 weeks were obtained from SPF Biotechnology (Beijing, China). Mice were administrated 20 µg/kg/d ISO for 7 days, and osmotic minipumps were implanted into the abdomens of the mice. Mice were randomly divided into 4 groups: the control group (mice administrated 0.9% NaCl), isoproterenol (ISO) group (mice administrated 20 µg/kg/d of ISO) ISO + Lv-NC group (mice administrated 2 × 10^9^ TU/ml, 50 μl Lv-NC), ISO + Lv H19 OE group (mice administrated ISO and 2 × 10^9^ TU/ml, 50 μl Lv-H19 OE). The recombinant lentivirus was injected into mice through the caudal vein. The mice were housed in 12-h light/dark cycle with free access to standard chow and tap water in a temperature-controlled room. When the experiment ended, the mice were euthanized by cervical dislocation, and myocardial and blood serum samples were collected for the following tests. Blood samples of mice (0.8–1.0 *ml*) was collected from the tail vein. The left ventricular ejection fraction, systolic dysfunction, posterior wall thickness, and fractional shortening were used to evaluate the CH. Then the collected left ventricular myocardial tissues were used for hematoxylin and eosin (HE) and Masson staining. This study was supervised and approved by the Ethics Committee of First Affiliated Hospital of Nanjing Medical University. The animal experiments were performed in accordance with the Guide for the Care and Use of Laboratory Animals.

### Ultrasound imaging measurements

Briefly, mice were anesthetized, and the prethoracic fur was removed using depilatory cream. Echocardiography was performed using the Mylab 30CV ultrasound system (Esaote S.P.A., Genoa, Italy) and a 10-MHz linear ultrasound transducer [23]. Finally, the left ventricular systolic diameter (LVsd) and left ventricular posterior wall diameter in diastole (LVPWd) were measured in at least three consecutive cardiac cycles. The ejection fraction (EF) and fractional shortening (FS) were calculated using the Vevo®2100 High-Resolution Imaging system (Visual Sonics). The formulae used for the evaluation of these parameter (EF% and FS%) were followed: EF = (EDV-ES)×100%/EDV, EDV: ventricular end diastolic volume; ES: end-systolic volume; FS = (Dd-Ds)/Dd×100%, Dd: left ventricular end-diastolic dimension, Ds: left ventricular end-systolic dimension.

### Cell culture and treatment

H9C2 cells were purchased from ATCC, USA. Cells were incubated in DMEM containing 10% FBS and 1% penicillin/streptomycin at 37°C with 5% CO_2_. Lv-lncRNA H19 and its corresponding control Lv-NC were purchased from Genebay (Nanjing, China) [24]. The miR-145-3p inhibitor, negative control oligonucleotide (NC inhibitor), miR-145-3p mimic and its corresponding control NC mimic and pcDNA3.1 SMAD and pcDNA3.1 vector were obtained from RiboBio (Guangzhou, China). Cells were transfected with pLKO.1/puro plasmids together with pCMV-dR8.91 and pCMV-VSV-G packing plasmids for 72 h. Then the lentiviral particle-enriched supernatant was collected and centrifuged. Cells were transduced with Lv lncRNA H19 or Lv NC at multiplicities of infection (MOI) of 20 overnight. For transfection, cells were treated with Lv-lncRNA H19, Lv-NC, miR-145-3p inhibitor, negative control (NC) inhibitor, miR-145-3p mimic, NC mimic, pcDNA3.1 or pcDNA3.1-SMAD4 with Lipofectamine 3000 (Invitrogen, USA) for 48 h in accordance with the manufacturer’s protocols. Cells were treated with ISO (20 μM) treatment for 48 h to induced CH development [25].

### Immunofluorescence staining

Cells were fixed 4 polychlorinated formaldehyde. After washing with 10% PBS for three times, the cells were incubated with a primary antibody against alpha-actinin and then with a secondary antibody. Subsequently, the cells were captured by a fluorescence microscope (Nikon, Japan).

### qRT-PCR

Total RNA was collected from cells and serum. Complementary DNA (cDNA) was synthesized with a cDNA synthesis kit (Roche, USA). qRT-PCR was conducted with SuperReal PreMix Plus (Tiangen, China). The expression levels were calculated with the 2^−ΔΔCt^ method. U6 and GAPDH were used as the loading controls. Each experiment was performed in triplicate. The primers for RT-qPCR are shown in supplementary materials in Table 2.

**Table 2.** Primer sequences used for qRT-PCR

Genes		Primer sequences (5’-3’)
Hsa-lncRNA H19	Forward	ACCACTGCACTACCTGACTC
	Reverse	CCGCAGGGGGTGGCCATGAA
Hsa-GAPDH	Forward	GGAGCGAGATCCCTCCAAAAT
	Reverse	GGCTGTTGTCATACTTCTCATGG
Mmu-lncRNA H19	Forward	GCTCCACTGACCTTCTAAAC
	Reverse	ACGATGTCTCCTTTGCTAAC
Mmu-ANP	Forward	GGAGCCTACGAAGATCCAGC
	Reverse	TTCGGTACCGGAAGCTGTTG
Mmu-BNP	Forward	GCTGTAACGCACTGAAGTTG
	Reverse	TCACTTCAAAGGTGGTCCCAG
Mmu-MYH7	Forward	TGGCAAGACGGTGACTGTGA
	Reverse	GGTTGACGGTGACGCAGAAG
Mmu-GAPDH	Forward	AGGTCGGTGTGAACGGATTTG
	Reverse	GGGGTCGTTGATGGCAACA
Rno-lncRNA H19	Forward	CGTTCCTTTAGTCTCCTGAC
	Reverse	AGTCCGTGTTCCAAGTCC
Rno-ANP	Forward	GAGCAAATCCCGTATACAGTGC
	Reverse	ATCTTCTACCGGCATCTTCTCC
Rno-BNP	Forward	GCTGCTGGAGCTGATAAGAGAA
	Reverse	GTTCTTTTGTAGGGCCTTGGTC
Rno-MYH7	Forward	GTGACGGTGGGAAAGGCAAAG
	Reverse	AAAGTGAGGATGGGTGGTCCT
Rno-GAPDH	Forward	CGCTAACATCAAATGGGGTG
	Reverse	TTGCTGACAATCTTGAGGGAG
Rno-miR-145-3p	Forward	GCCCTGTAGTGTTTCCTACTT
	Reverse	GTGCAGGGTCCGAGGT
Rno-SMAD4	Forward	GGTGGCTGGTCGGAAAGG
	Reverse	CGTGGGTAAGGATGGCTGT
Rno-U6	Forward	CTCGCTTCGGCAGCACA
	Reverse	AACGCTTCACGAATTTGCGT

### Western blot

Total protein was obtained from serum samples, tissues and cells. 30 mg of mice left ventricular myocardium was used to extract protein. The protein concentration was calculated with a BCA Kit (Bio-Rad, USA). A total of 20 μg of protein was separated by 12% sodium dodecyl sulfate polyacrylamide gel electrophoresis (SDS-PAGE). Then, the protein was transferred onto membranes. After blocking with 5% skimmed milk, the membranes were incubated with primary antibodies and then with secondary antibodies. Finally, the protein bands were captured with an E-Gel imaging system (Thermo Fisher Scientific, USA), and calculated with Quantity One Software (Bio-Rad, USA). The primary antibodies used in these experiments were as follows: SMAD4 (sc-7966), atrial natriuretic peptide (ANP, sc-515,701); brain natriuretic peptide (BNP, sc-271,185); MYH7 (sc-53,089); SMAD4 (sc-7966); and GAPDH (sc-47,724), all purchased from Santa Cruz Biotechnology Inc. Image J software (version 1.8.0) was used to analyze the gray value of the WB.

### Subcellular localization assay

To determine the cellular localization of H19, the nuclear fraction was isolated from the cytoplasm using a Nuclear and Cytoplasmic Protein Extraction Kit (Beyotime, Shanghai, China) according to the manufacturer’s protocol. The expression patterns of H19, U6, and GAPDH in nuclear and cytoplasmic fractions were analyzed using qRT-PCR assay.

### Luciferase reporter assay

The online databases DIANA and TargetScan were used to predict the targets of H19 and miR-145-3p, respectively [26]. Luciferase reporter plasmids were synthesized and provided by GenePharama, Shanghai. Then cells were cotransfected with H19 3ʹuntranslated region (3ʹUTR) wild type (WT) or H19 3ʹUTR mutant (MUT), and miR-145-3p mimic or NC mimic with Lipofectamine 3000. The results were determined with a Dual-Lucifer Reporter Assay System (Promega, Madison, WI, USA) [27].Renilla luciferase was used as loading control. For miR-145-3p and SMAD4, cells were cotransfected with miR-145-3p mimic or NC mimic and SMAD4 3ʹUTR WT or SMAD4 3ʹUTR MUT as described above.

### Statistical analysis

All data are presented as the mean ± SD and analyzed with SPSS 19.0. Two-tailed Student’s *t* test was utilized to evaluate the difference between two groups. The differences among multiple groups were analyzed by one-way ANOVA followed by Tukey’s test. The Pearson method was performed for correlation analysis. *P *< 0.05 was considered statistically significant.

## Results

In this study, we explored the roles of H19 in CH *in vivo* and *in vitro* models induced by ISO. MiRNAs microarray analysis showed that the expression levels of miR-145-3p were significantly upregulated in patients with AMI. SMAD4 was predicted and proven to be a target of miR-145-3p. H19/miR-145-3p/SMAD4 was proved to be a promising therapeutic target for CH.

### H19 is decreased in CHF patients

**Figure 1.**
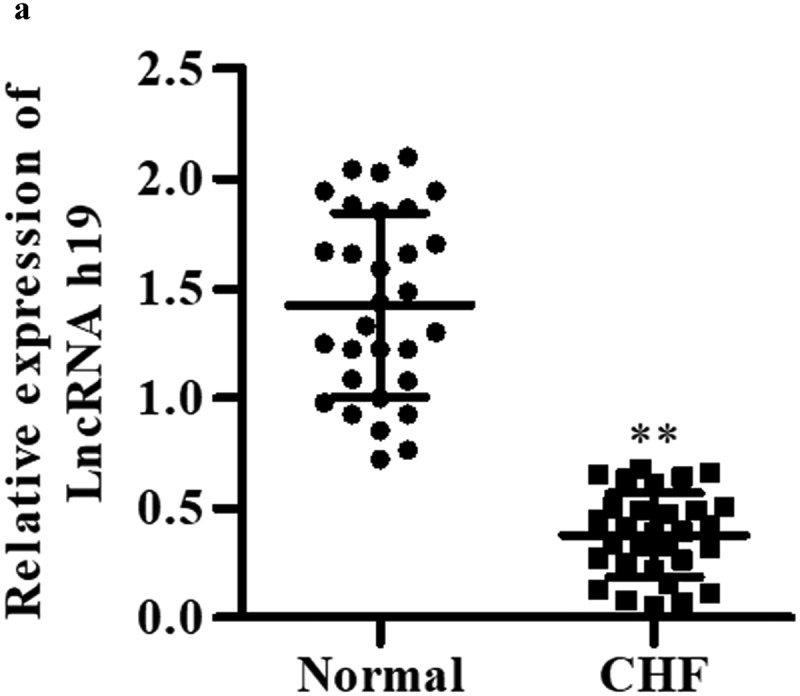
**H19 is decreased in CHF patients**. The serum level of H19 determined by qRT-PCR. All data were presented as mean ± SD. **P < 0.01 vs. normal group.

qRT-PCR was performed to determine the serum level of H19 in clinical samples. As shown in Figure 1, the serum level of H19 was significantly decreased in CHF patients compared with healthy controls, suggesting that H19 may play an important role in the progression of CHF.

### The overexpression of H19 ameliorates ISO-induced cardiac hypertrophy in vivo

**Figure 2.**
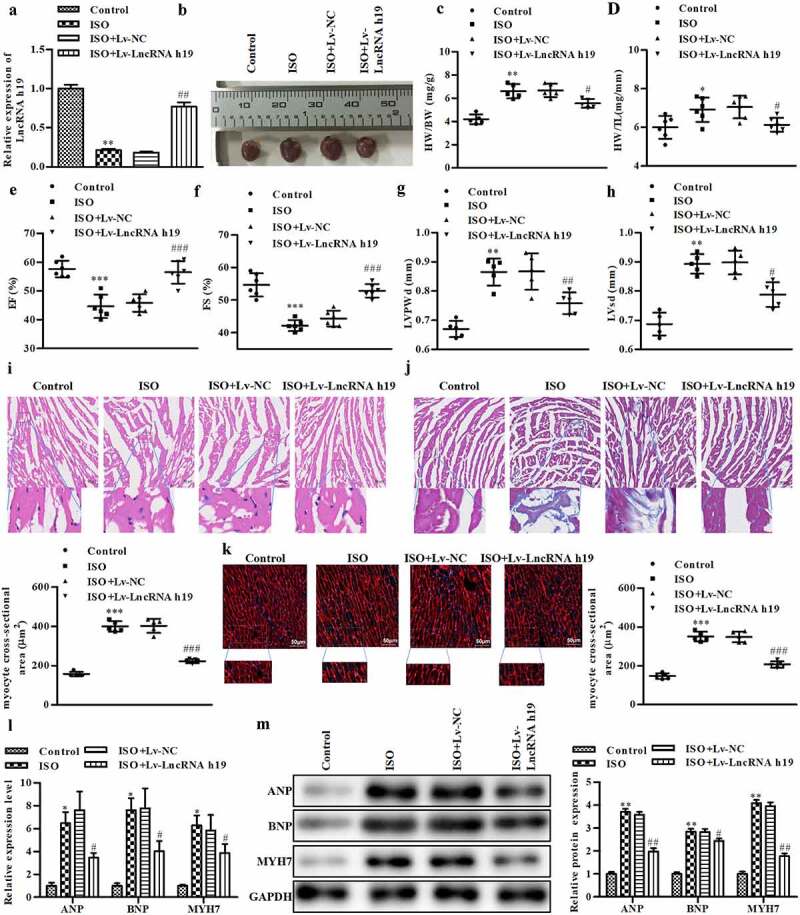
**The overexpression of H19 inhibits CH in vivo**. (a) The expression level of H19 was calculated by qRT-PCR. (b) Heart size of mice. (c) Heart weigh vs. body weight. (d) Heart weight vs. tibial length. (e) Left ventricular ejection fraction. (f) Left ventricle fraction shortening. (g) Left ventricular posterior wall thickness. (h) Left ventricular systolic dysfunction. (i) Cardiac hypertrophic growth determined by HE staining. (j) Cardiac hypertrophic growth determined by masson staining. (k) The histology of cardiomyocytes hypertrophy determined by wheat germ agglutinin staining. Panel I, J, K top and bottom: Magnifications, ×10 and 40, respectively. Scale bar = 50 µm. (l) The mRNA level of ANP, BNP, and MYH7 were determined by qRT-PCR. (m) The protein level of ANP, BNP, and MYH7 were detected by Western blot. Control group: mice administrated with 0.9% NaCl, ISO group: mice administrated with ISO, ISO + Lv NC group: mice administrated with ISO and Lv-LncRNA NC OE, ISO + Lv-LncRNA H19 OE group: mice administrated with ISO and Lv-LncRNA H19 OE. n = 5. All data were presented as mean ± SD. *P < 0.05, **P < 0.01, ***P < 0.001 vs. control group. ^#^*P *< 0.05, ^##^*P *< 0.01, ^###^*P *< 0.001 vs. ISO + Lv-NC group.

To explore the potential roles of H19 in CH, mice were treated with ISO to construct CH i*n viv*o model. First, we obtained the representative M mode images shown in Figure S1 to evaluate cardiac function. As shown in Figure 2a, transfection with Lv-LncRNA H19 restored the ISO-induced decrease in H19 expression. Moreover, the heart size was significantly increased in mice administrated ISO and was decreased after the treatment with Lv-LncRNA H19 (Figure 2b). Moreover, ISO administration significantly increased mouse heart weight. However, the treatment of Lv-LncRNA H19 significantly decreased the heart weight (Figure 2c and d). The left ventricular ejection fraction in mice exposed to ISO was significantly decreased, while this was reversed by Lv-LncRNA H19 (Figure 2e). Moreover, Lv-LncRNA H19 treatment inhibited the decrease in left ventricle fractional shortening induced by ISO (figure 2f). Additionally, the left ventricular posterior wall thickness was increased in mice exposed to ISO, which was ameliorated by Lv-LncRNA H19 (Figure 2g). ISO induced left ventricular systolic dysfunction, which was restored by Lv-LncRNA H19 (Figure 2h). Moreover, the results of the histological assay showed that ISO administration induced CH, which was suppressed by Lv-LncRNA H19 (Figure 2i, 2j, 2k). Furthermore, the biomarkers of CH were measured. As shown in Figure 2l, 2m, ISO significantly increased the expression of ANP, BNP, and MYH7 at the mRNA and protein levels, which was repressed by Lv-LncRNA H19 [28,29].

### Overexpression of H19 inhibits ISO-induced CH development in vitro

**Figure 3.**
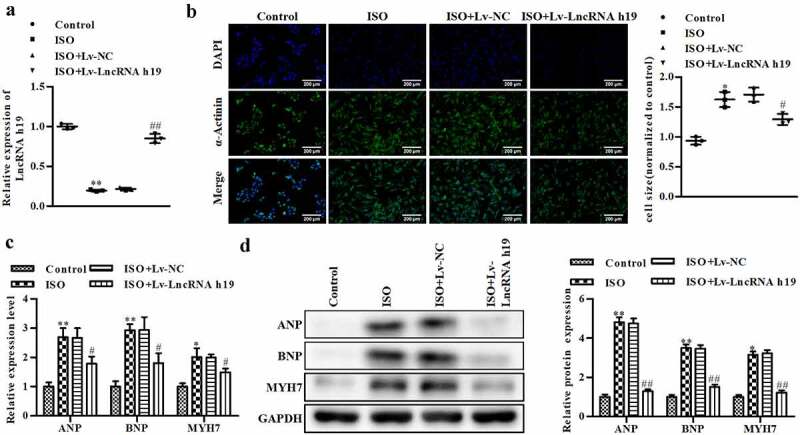
**The overexpression of H19 inhibits CH in vitro**. (a) The RNA level of H19 was determined by qRT-PCR. (b) The level of α-actinin was determined by immunofluorescence assay, magnifications, ×40. (c) The mRNA level of ANP, BNP, and MYH7 were determined by qRT-PCR. (d) The protein level of ANP, BNP, and MYH7 were detected by Western blot. Control group: cells treated without ISO, ISO group: cells treated with ISO, ISO + Lv NC group: cells treated with ISO and * NC OE, ISO + * H19 OE group: cells treated with ISO and * H19 OE. n = 3. All data were presented as mean ± SD. *P < 0.05, **P < 0.01 vs. control group. ^#^*P *< 0.05, ^##^*P *< 0.01 vs. ISO + Lv-NC group.

To further investigate the roles of H19 in CH, we overexpressed H19 in ISO-treated H9C2 cells. As shown in Figure 3a, Lv-LncRNA H19 significantly increased the level of H19 in H9C2 cells compared with the control group (Figure 3a). Moreover, the increase in cell size (superficial area of cardiomyocytes) induced by ISO was alleviated by Lv-LncRNA H19 (Figure 3b). Moreover, ISO exposure increased the mRNA and protein levels of ANP, BNP, and MYH7, which were suppressed by Lv-LncRNA H19 (Figure 3c and d).

### miR-145-3p is a target of H19

**Figure 4.**
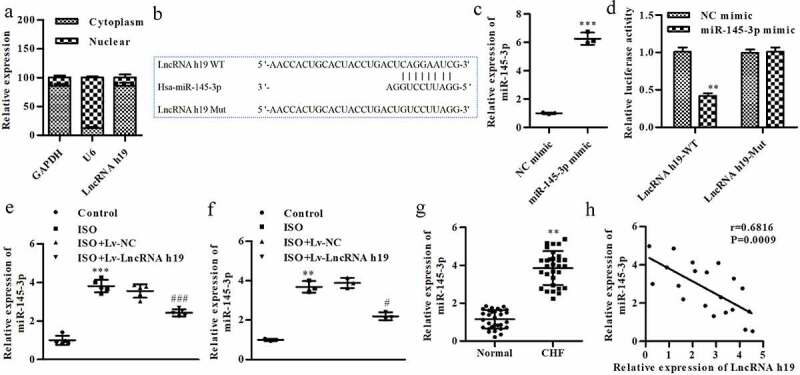
**miR-145-3p is a target of H19**. (a) The enrichment of H19 calculated by subcellular localization assay. (b) The binding site of miR-145-3p on H19. (c) The expression of miR-145-3p was determined by qRT-PCR, ***P < 0.001 vs. NC mimic. (d) miR-145-3p was proved to be a target of H19, ***P < 0.01 vs. NC mimic. (e) The expression level of miR-145-3p in mice cardio tissues, ***P < 0.001 vs. Control group. ^#^*P *< 0.05 vs. ISO + Lv-NC group. (f) The expression of miR-145-3p in cells, **P < 0.01 vs. control group. ^#^*P *< 0.05 vs. ISO + Lv-NC group. (g) The level of miR-145-3p in CHF patients, **P < 0.01 vs. normal group. (h) The correlation analyzed by Pearson methods. Control group: cells treated without ISO, ISO group: cells treated with ISO, ISO + Lv-NC group: cells treated with ISO and Lv-LncRNA NC OE, ISO + Lv-LncRNA H19 OE group: cells treated with ISO and Lv-LncRNA H19 OE, NC mimics group: cells were treated with NC mimic, miR-145-3p mimic group: cells were treated with miR-145-3p mimic. n = 3. All data were presented as mean ± SD.

To investigate the underlying molecular mechanisms, we hypothesized that H19 may regulate the progression of CH by regulating its target miRNA. Subcellular localization assays revealed that H19 was significantly enriched in the cytoplasmic fraction of H9C2 cells (Figure 4a). Bioinformatics analysis showed that miR-145-3p was a target of H19, since miR-145-3p participated broadly in heart diseases (Figure 4b). The expression of miR-145-3p was significantly increased after transfection with miR-145-3p mimic, suggesting that cells were successfully transfected (Figure 4c). Additionally, cotransfection of H19 3ʹUTR WT and miR-145-3p mimic significantly decreased the luciferase activity (Figure 4d). ISO treatment significantly increased miR-145-3p levels *in vitro* and *in vivo*, which was reversed by Lv-LncRNA H19 (Figure E and F). Moreover, the expression of miR-145-3p was increased in CHF patients compared with healthy groups (Figure 4g). The expression of miR-145-3p was negatively correlated with H19 (Figure 4h).

### Knockdown of miR-145-3p promotes the cardioprotective role

**Figure 5.**
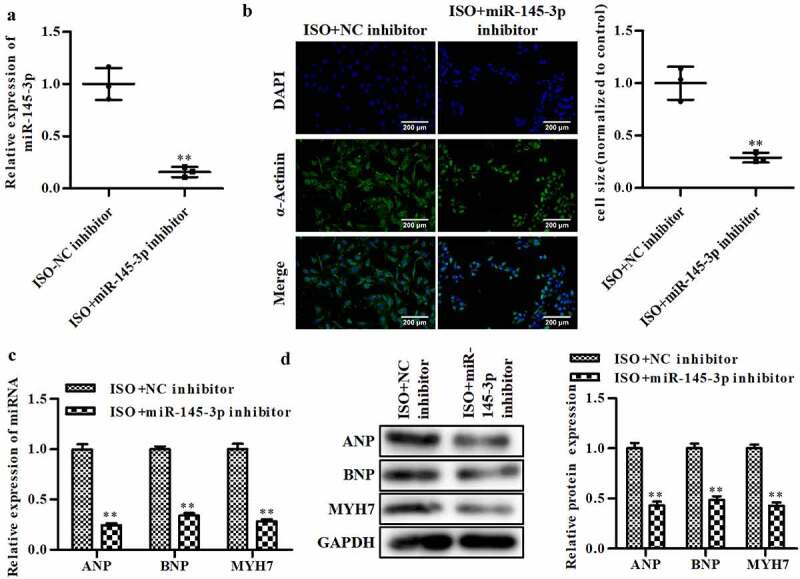
**Knockout of miR-145-3p facilitates CH in vitro**. (a) The level of miR-145-3p was determined by qRT-PCR. (b) The level of α-actinin was determined by immunofluorescence assay, magnifications, ×40. (c) The mRNA level of ANP, BNP, and MYH7 were determined by qRT-PCR. (d) The protein level of ANP, BNP, and MYH7 were detected by Western blot. ISO-NC inhibitor group: cells were treated with ISO and NC inhibitor, ISO + miR-145-3p inhibitor group: cells were treated with ISO and miR-145-3p inhibitor. n = 3. All data were presented as mean ± SD. **P < 0.01 vs. ISO+NC inhibitor.

To verify the possible roles of miR-145-3p in the progression of CH, qRT-PCR was performed to determine the level of miR-145-3p. As shown in Figure 5a, the expression of miR-145-3p was significantly decreased after transfection with the miR-145-3p inhibitor. Knockout of miR-145-3p significantly decreased cell size (Figure 5b). Furthermore, inhibit miR-145-3p significantly suppressed ANP, BNP, and MYH7 expressions at the mRNA and protein levels (Figure 5c and d).

### H19 regulates SMAD4 by sponging miR-145-3p

**Figure 6.**
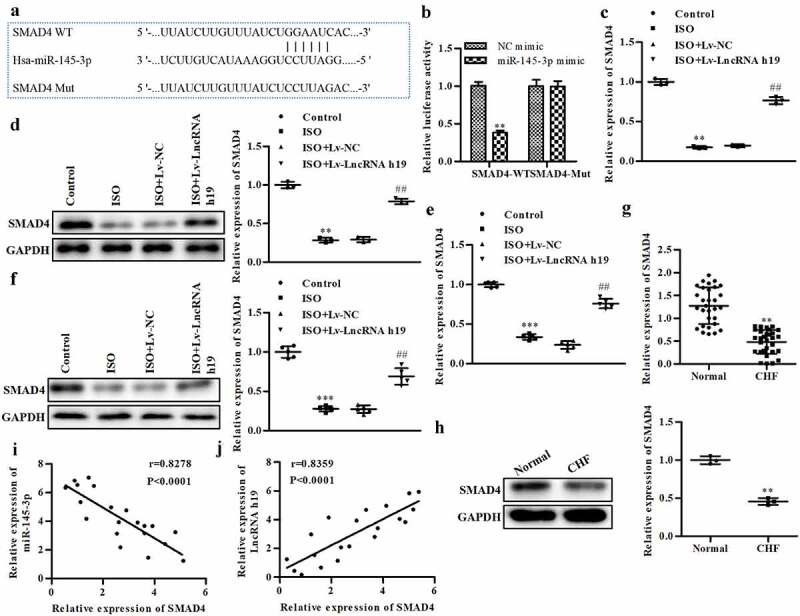
**H19 regulates SMAD4 via sponging miR-145-3p**. (a) SMAD4 was a target of miR-145-3p. (b) The luciferase reporter assay, **P < 0.01 vs. NC mimic. (c) The mRNA level of SMAD4 in cells, **P < 0.01 vs. control. ^##^*P *< 0.01 vs. ISO+Lv-NC. (d) The protein level of SMAD4 in cell, **P < 0.01 vs. control. ^##^*P *< 0.01 vs. ISO+Lv-NC. (e) The mRNA level of SMAD4 in left ventricular myocardium of mice. ***P < 0.001 vs. control. ^##^*P *< 0.01 vs. ISO+Lv-NC. (f) The protein level of SMAD4 in mice tissues, ***P < 0.001 vs. Control. ^##^*P *< 0.01 vs. ISO+Lv-NC. (g) The mRNA level of SMAD4 determined by qRT-PCR in CHF, **P < 0.01 vs. normal. (h) The protein level of SMAD4 in CHF, **P < 0.01 vs. normal. (i) The correlation between SMAD4 and H19 evaluated by Pearson analysis. (j) The correlation between SMAD4 and miR-145-3p evaluated by Pearson analysis. n = 3. All data were presented as mean ± SD. image J software (version 1.8.0) was used to analyze the gray value of the WB and the bar graphs showed quantitative data results.

SMAD4 deficiency contributes to the initiation and development of CH. To explore the underlying molecular mechanisms, we examined the roles of SMAD4 in CH. SMAD4 was predicted and proven to be a target of miR-145-3p (Figure 6a and b). The decrease in SMAD4 induced by ISO was reversed by Lv-LncRNA H19 at both the mRNA and protein levels *in vivo* and *in vitro* (Figure 6c-f). Moreover, SMAD4 was reduced in CHF patients (Figure 6g and h). The expression of SMAD4 was positively correlated with H19 but negatively correlated with miR-145-3p (Figure 6i and j). We also detected changes in H19, miR-145-3p and SMAD4 levels in the serum of the mouse model, as shown in Figure 6k, and found that the expression of H19 and SMAD4 was decreased in the ISO group, while that of miR-145-3p was increased.

### Overexpressed SMAD4 facilitates the effects of H19 on CH

**Figure 7.**
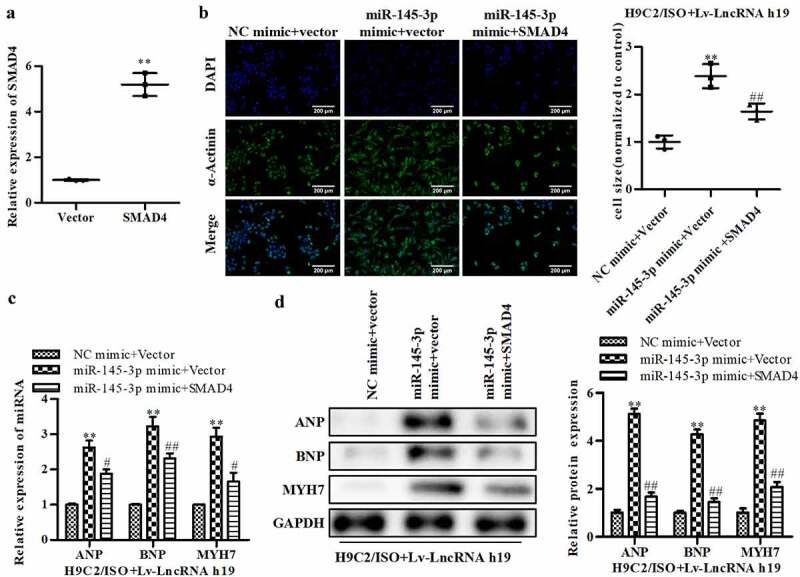
**Overexpressed SMAD4 facilitates the inhibitory effects of H19 on CH**. (a) The level of SMAD4 was determined by qRT-PCR. **P < 0.01 vs. Vector. (b) The level of α-actinin was determined by immunofluorescence assay, Magnifications, ×40. (c) The mRNA level of ANP, BNP, and MYH7 were determined by qRT-PCR. (d) The protein level of ANP, BNP, and MYH7 were detected by Western blot. NC mimic + vector group: cells treated with ISO, NC mimic and vector. miR-145-3p mimic + vector group: cells treated with ISO, miR-145-3p mimic and vector. miR-145-3p mimic + SMAD4 group: cells treated with ISO, miR-145-3p mimic and SMAD4. n = 3. All data were presented as mean ± SD. **P < 0.01 vs. NC mimic + vector. ^#^*P *< 0.05, ^##^*P *< 0.01 vs. miR-145-3p mimic + vector.

To verify the potential roles of SMAD4 in CH, qRT-PCR was conducted to calculate the expression of SMAD4. Cells treated with ISO and Lv-LncRNA H19 were used in further studies. The expression of SMAD4 was significantly increased in pcDNA3.1-SMAD4-treated cells, which suggested that the cells were successfully transfected (Figure 7a). The overexpression of SMAD4 abated the increase in cell size induced by miR-145-3p (Figure 7b). Additionally, SMAD4 reversed the increase in ANP, BNP, and MYH7 induced by miR-145-3p at both the mRNA and protein levels, and this effect was augmented by SMAD4 (Figure 7c and d).

## Discussion

In this study, H19 was shown to play a regulatory role in CH. H19 was reduced in CHF patients and in mice with CH induced by ISO *in vivo*, and its overexpression protected heart function against CH. Additionally, H19 acted as a ceRNA by sponging miR-145-3p. Overexpressed H19 induced the suppression of miR-145-3p, promoting the activation of SMAD4 (a target of miR-145-3p). The increase in SMAD4, a crucial regulator of CH, inactivated CH biomarkers, such as ANP, BNP, and MYH7. Thus, H19 played a cardioprotective role and a new anti-CH signaling pathway was elucidated, the H19/miR-145-3p/SMAD4 axis. This sheds new light on the roles of lncRNAs in CHF.

Accumulating evidences has revealed that lncRNAs play a crucial role in the initiation and progression of CVD [30]. However, only a few lncRNAs function as anti-CH in CVD. Here, H19 serves as a CH suppressor [31]. First, overexpression of H19 decreased cell size, and the levels of ANP, BNP and MYH7, which intensively participate in CH. Furthermore, the overexpression of H19 alleviated the extent and development of CH induced by ISO and enhanced heart function *in vivo*. These findings suggest that H19 plays a cardioprotective role and suppresses CH development and progression both *in vitro* and *in vivo*. Recent studies have demonstrated that H19 inhibits the development of CVD [32].H19 interacts with miR-130a-3p and miR-17-5p to modify the radioresistance and chemosensitivity of cardiac carcinoma cells [33], and protects against acute myocardial infarction by activating autophagy in mice [34]. Targeted inhibition of long non-coding RNA H19 blocks anaplastic thyroid carcinoma growth and metastasis [35] and expression of long non-coding RNA H19 predicts distant metastasis in minimally invasive follicular thyroid carcinoma [36]. In addition, Choong *et al* found that H19 and its interacting protein YB-1 are crucial for extracellular matrix regulation during cardiac remodeling [37]. Although H19 inhibits myocardial ischemia-reperfusion injury and reduces the increase in cell size and prohypertrophic gene levels, the mechanisms by which H19 inhibits CH have not been fully elucidated [38]. H19 may have different regulatory patterns and functions in CVD. Advances in gene therapy make H19-targeted delivery possible for clinical CH therapy.

Collective evidence has revealed that lncRNAs function as ceRNAs and regulate the initiation and progression of CVD by targeting miRNAs and regulating chromosome remodeling, transcriptional control and RNA degradation [39]. Moreover, aberrantly expressed H19 is associated with various heart diseases and is suitable for use as a biomarker for CH [40]. In this study, miR-145-3p was predicted and proven to be a target of H19 bioinformatics analysis and luciferase reporter assay. Specifically, the expression of miR-145-3p was negatively correlated with H19. MiR-145-3p is diagnostic biomarker for acute myocardial infarction, and regulates the fibrosis response [18]. MicroRNA-145 overexpression inhibits neuroblastoma tumorigenesis and Phospholipase D 5 (PLD5) to downregulate cell proliferation and metastasis to mitigate prostate cancer [41,42]. A previous study demonstrates that miR-145 is involved in microvascular development, pericyte function and disease progression and collectively participates in the initiation and progression of CH [43]. In the present study, miR-145-3p was increased in CHF patients and in ISO-treated cells. These results suggested that miR-145-3p was associated with the progression of CH. However, the decrease in miR-145-3p promoted the suppression of ANP, BNP, and MYH7. Therefore, miR-145-3p may play a positive role in the progression of CH.

MiRNAs function as posttranscriptional regulators by binding to the 3ʹUTR of their targets. In this study, SMAD4 was proven to be a target of miR-145-3p. SMAD4 was decreased in CHF patients and CH model animals. The expression of SMAD4 was positively correlated with H19, while negatively correlate with miR-145-3p. Additionally, miR-145-3p restored SMAD4 to normal levels, which revealed that H19 regulated SMAD4 by sponging miR-145-3p. H19 activated SMAD4 *in vivo* and *in vitro*. Interestingly, the depletion of SMAD4 in cardiomyocytes contributes to cardiac hypertrophy [44]. SMAD signaling induces cardiomyocyte apoptosis, which may protect against CH [45]. Overall, H19 could increase the expression of SMAD4. However, we did not show the reverse effects of silencing SMAD4 on H19 overexpression. We acknowledge that as a limitation of this study. Subsequent studies should further validate these findings by silencing SMAD4. In this study, SMAD4 overexpression facilitated H19-induced inactivation of ANP, BNP, and MYH7 and decreased cell size. These results suggested that SMAD4 suppressed the progression of CH, which was consistent with the latest study [46]. Therefore, the H19/miR-145-3p/SMAD4 axis may be a novel biomarker for CH.

## Conclusions

H19 was decreased in CHF patients and CH cells. The overexpression of H19 inhibited the progression of CH. H19 induced an increase in SMAD4 by sponging miR-145-3p, which further repressed the growth of CH. This may provide a novel therapy for CH.

## Supplementary Material

Supplemental Material

## Data Availability

The data that support the findings of this study are available from the corresponding author upon reasonable request.

## Highlights:

- We demonstrated H19 could participate in regulating ISO-induced CH development both *in vivo* and *in vitro*.
- It was predicted by online databases that MiR-145-3p was a target of H19 and SMAD4 was a target of miR-145-3p, which was verified by Luciferase assays.
- The H19 overexpression resulted in the suppression of cardiac hypertrophy and inhibited protein expression levels of CH markers.
- H19/miR-145-3p/SMAD4 axis should be a negative regulator involved in CH progression.

## Declarations

*Ethics approval and consent to participate*

The study was approved by the Ethics Committee of First Affiliated Hospital of Nanjing Medical University Hospital. Each patient signed the informed consent. The contents of this study are under full compliance with government policy and the Declaration of Helsinki.
