# Supplementary figures and images for: Long noncoding RNA H19 suppresses cardiac hypertrophy through the MicroRNA-145-3p/SMAD4 axis
Source: Bioengineered. 2022 Feb 9;13(2):3826–39. doi: 10.1080/21655979.2021.2017564 (PMC8973863; doi:10.1080/21655979.2021.2017564)

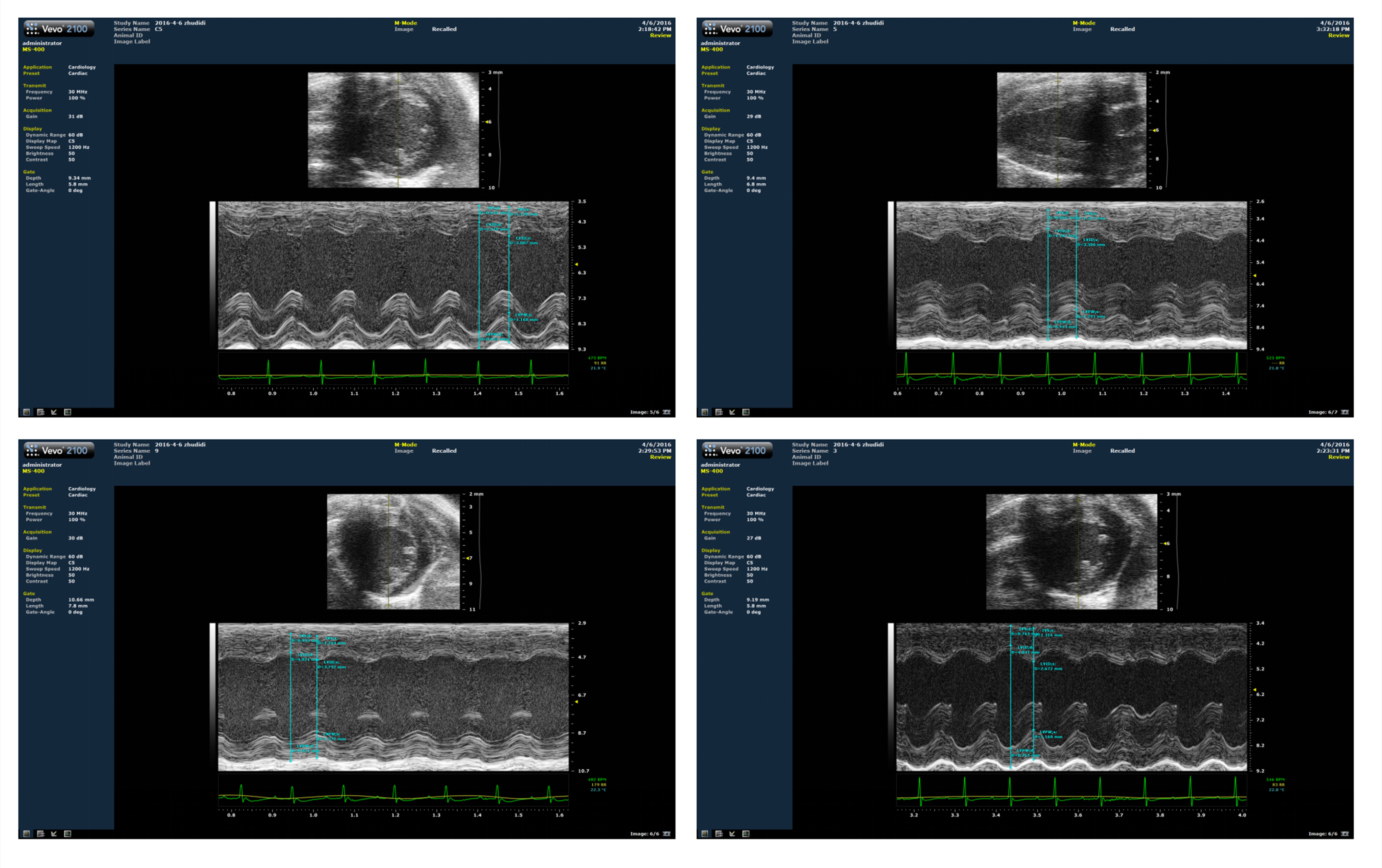

Supplement: Supplemental Material [file KBIE_A_2017564_SM7971.tif]
